# The Effects of Landmark Salience on Drivers’ Spatial Cognition and Takeover Performance in Autonomous Driving Scenarios

**DOI:** 10.3390/bs15070966

**Published:** 2025-07-16

**Authors:** Xianyun Liu, Yongdong Zhou, Yunhong Zhang

**Affiliations:** 1 Faculty of Psychology, Tianjin Normal University, Tianjin 300387, China; rubia@stu.tjnu.edu.cn; 2 Key Research Base of Humanities and Social Sciences of the Ministry of Education, Academy of Psychology and Behavior, Tianjin Normal University, Tianjin 300387, China; 3 Key Laboratory of Human Factors and Ergonomics, State Administration for Market Regulation, China National Institute of Standardization, Beijing 100191, China

**Keywords:** autonomous driving, landmark, visual salience, structural salience, spatial cognition, wayfinding, takeover

## Abstract

With the increasing prevalence of autonomous vehicles (AVs), drivers’ spatial cognition and takeover performance have become critical to traffic safety. This study investigates the effects of landmark salience—specifically visual and structural salience—on drivers’ spatial cognition and takeover behavior in autonomous driving scenarios. Two simulator-based experiments were conducted. Experiment 1 examined the impact of landmark salience on spatial cognition tasks, including route re-cruise, scene recognition, and sequence recognition. Experiment 2 assessed the effects of landmark salience on takeover performance. Results indicated that salient landmarks generally enhance spatial cognition; the effects of visual and structural salience differ in scope and function in autonomous driving scenarios. Landmarks with high visual salience not only improved drivers’ accuracy in making intersection decisions but also significantly reduced the time it took to react to a takeover. In contrast, structurally salient landmarks had a more pronounced effect on memory-based tasks, such as scene recognition and sequence recognition, but showed a limited influence on dynamic decision-making tasks like takeover response. These findings underscore the differentiated roles of visual and structural landmark features, highlighting the critical importance of visually salient landmarks in supporting both navigation and timely takeover during autonomous driving. The results provide practical insights for urban road design, advocating for the strategic placement of visually prominent landmarks at key decision points. This approach has the potential to enhance both navigational efficiency and traffic safety.

## 1. Introduction

### 1.1. Research Background

Autonomous vehicles (AVs) are rapidly ascending in importance, dramatically improving vehicle active safety and energy efficiency (Kumsa Kurse et al., 2024). These advancements contribute to a cascade of benefits, including reduced emissions, an increase in traffic infrastructure capacity, and, importantly, improved accessibility for both the elderly and individuals with disabilities (Ahmed et al., 2022; Fagnant & Kockelman, 2015; Robertson et al., 2019). The influence of AVs on individual lives, however, varies depending on the specific level of vehicle automation. To provide a clear framework for understanding these nuances, the Society of Automotive Engineers (SAE) has developed a classification system. This system categorizes autonomous driving systems (ADS) into six distinct levels, ranging from L0 to L5. Crucially, each ascending level represents a corresponding increase in the vehicle’s intelligence and, consequently, its degree of automation. Currently, most automotive companies are transitioning from partial (L2) to conditional driving (L3) automation and are moving towards advanced and fully automated driving (L4 and L5) (Tan & Zhang, 2025). At L2, driving operations are handled by the system, but the driver must monitor the environment and take over when necessary. At L3, both driving operations and environmental monitoring are managed by an automated system, but the driver must immediately take over in emergencies and respond to unexpected situations. Notably, during L3 autonomous driving, drivers do not need to constantly supervise the vehicle and can engage in various non-driving-related tasks (NDRTs), such as reading, watching videos, browsing the internet, or making calls (Dillmann et al., 2021; Shi & Frey, 2021).

Engagement in NDRTs, however, can impair drivers’ spatial cognition, which is critical for effective and timely takeover. A decline in spatial cognition adversely affects the driver’s situational awareness and reaction time, thereby increasing the risk of traffic incidents during transitions from automated to manual control. Consequently, even though driving responsibilities are largely assumed by the automated system at L2 and L3, drivers must remain alert and capable of promptly responding to a Take-Over Request (TOR) to ensure safety. As AVs become more prevalent, the cognitive demands placed on drivers during control transitions—particularly their spatial cognition and takeover performance—have emerged as key factors influencing traffic safety. Therefore, advancing research aimed at enhancing drivers’ spatial cognition and reducing takeover response time in automated driving contexts has become a pressing priority in the field of autonomous driving.

### 1.2. Literature Review

#### 1.2.1. How Does Driving Performance Differ Between Autonomous and Manual Driving?

Compared to traditional driving, drivers’ cognitive performance under autonomous driving conditions exhibits significant differences. On the one hand, autonomous driving negatively impacts drivers’ cognition, mainly in the following respects: (1) attention diversion: drivers pay significantly less attention to the road ahead under autonomous driving compared to manual driving and often engage in non-driving-related tasks (NDRTs) (Pipkorn et al., 2022; Tivesten et al., 2019; Victor et al., 2005). (2) reduced vigilance: drivers’ perception of danger under autonomous driving significantly diminishes (Greenlee et al., 2018), while this decline is less pronounced or non-existent in manual driving (Parasuraman & Nestor, 1991); (3) deterioration in driving performance: simulator studies have found that drivers’ reaction speeds to emergencies are significantly slower under autonomous driving than in manual driving, especially when drivers are engaged in secondary tasks that require visual attention (Bianchi Piccinini et al., 2015). On the other hand, autonomous driving may enhance certain aspects of drivers’ spatial cognition. Studies by Cheng (2022) and Xu (2021) indicate that drivers’ spatial memory is stronger under autonomous driving compared to traditional driving. However, Xu (2021) also notes that the addition of takeover processes in autonomous driving scenarios may negatively affect drivers’ spatial memory. Overall, the mechanisms influencing drivers’ spatial cognition and takeover behavior in autonomous driving scenarios remain unclear, with most studies focusing on individual driver effects and neglecting environmental factors.

#### 1.2.2. Autonomous Driving and Takeover

Timely takeover of a vehicle during emergencies is crucial for safe autonomous driving. Takeover is defined as the process by which the driver quickly searches for environmental information, formulates an operation plan, selects the best option, and executes the corresponding action (Gold et al., 2015). Many factors influence takeover, including the form of the takeover request (Haas & van Erp, 2014), takeover lead time (Mok et al., 2015; Wu et al., 2019), non-driving-related tasks (Horrey et al., 2017), and driver fatigue (Guo et al., 2021). A reasonable takeover lead time helps participants better handle emergencies. However, there is considerable debate regarding the optimal takeover lead time. Previous studies compared relatively short takeover lead times (e.g., 5 s and 6 s) (Gold et al., 2013) with longer ones (e.g., 7 s and 8 s) (Wandtner et al., 2018) to determine the minimum takeover lead time required for safe takeover. Some studies suggest that a longer takeover lead time may be more appropriate (Eriksson & Stanton, 2017; Radlmayr & Bengler, 2015). Therefore, this study adopts a 7 s takeover lead time setting.

Given the complex mental activities involved in actual takeover, researchers have proposed several takeover models to simplify the takeover process (Heikoop et al., 2016; Zeeb et al., 2015). Heikoop et al. (2016) focus on analyzing the psychological structure of the takeover process, while Zeeb et al. (2015) introduce eye movement indicators to propose a takeover model considering visual distraction. Notably, in addition to driver factors and the form and lead time of the takeover request, the driving environment also significantly impacts takeover (Gold et al., 2016; Radlmayr et al., 2014). While a substantial portion of research incorporates weather conditions (sunny skies, dense fog, driving snow, and pouring rain) and road types (from bustling urban roads and expansive highways to winding curves and the subtle distinctions between marked and unmarked lanes) into their experimental designs, a surprising scarcity of studies directly investigates the impact of these very factors on takeover performance (Li et al., 2018; Louw et al., 2015, 2017). This oversight is particularly striking given the mounting evidence suggesting that the surrounding environment exerts a powerful influence on driver behavior (Megías et al., 2011; Walker & Trick, 2019).

#### 1.2.3. Autonomous Driving and Landmarks

Related research has demonstrated that the surrounding environment affects driving behavior. Emotionally charged billboards on the roadside can influence drivers’ attention and braking decisions (Megías et al., 2011; Walker & Trick, 2019), and billboards are common traffic landmarks in the driving environment. Landmarks are objects that navigators tend to use due to their distinctive features (Caduff & Timpf, 2008). These objects are noticed by navigators because they possess attributes that stand out from the surrounding environment, known as landmark salience (Hamburger & Röser, 2014). Visual salience has long been considered the primary feature that defines and is useful for landmark selection. Sorrows and Hirtle (1999) divided landmark salience into three aspects: visual salience, cognitive salience, and structural salience. Specifically, visual salience refers to the landmark being visually distinctive (e.g., due to its color, size, shape, or visibility). For instance, a large building or an unusually shaped structure might be visually salient. Cognitive salience refers to the landmark’s historical or cultural significance (e.g., the Statue of Liberty). Structural salience refers to landmarks located at easily accessible points (e.g., a sign at a street intersection). These characteristics enable landmarks to be effectively used in wayfinding tasks. Both structural and visual attributes of landmarks have been analyzed in prior research, with findings indicating that each plays a significant role in influencing wayfinding behavior. Visual characteristics such as visibility and color have been considered the primary features defining and useful for landmark selection. With respect to structural characteristics (landmark placement), research has shown that landmarks located on routes and particularly those positioned at decision points—locations where directional changes occur—are more likely to be noticed, remembered, and used by individuals during navigation. In contrast, off-route landmarks tend to be less effective. Landmarks are crucial for individual navigation, providing information on when and where route changes occur, serving as important reference points for navigating the environment and finding routes to destinations (Michon & Denis, 2001).

Landmark knowledge is one of the primary and most essential types of spatial knowledge individuals acquire when exploring new environments. It is crucial for establishing routes and obtaining survey knowledge (Siegel & White, 1975). During navigation, the relationship between landmarks and spatial cognition is often associated with turning directions, with participants showing a strong preference for landmarks at turning points (Röser et al., 2011, 2012). Many studies emphasize the importance of turning point landmarks (Janzen, 2006; Janzen & van Turennout, 2004), but some research also highlights the role of landmarks on the sides of the road (Lovelace et al., 1999; Tlauka & Wilson, 1994). Therefore, it is important to consider both roadside landmarks and turning point landmarks (Yesiltepe et al., 2021). It is well known that the increased use of GPS navigation has led to a decline in individuals’ spatial cognition skills, which may further affect independence, autonomy, and quality of life. Although drivers have become accustomed to following GPS navigation, landmarks still serve as visual reference points during driving, thereby enhancing drivers’ understanding of their spatial environment. This not only helps improve drivers’ memory of routes but also provides directional guidance at complex turns or intersections, effectively enhancing drivers’ spatial cognition. However, as mentioned earlier, although visually salient landmarks like billboards play a significant role in capturing drivers’ attention (Megías et al., 2011; Walker & Trick, 2019), their excessive attraction may distract drivers at non-decision points. Therefore, the interactive effects of visual and structural salience on drivers warrant further study.

### 1.3. Research Questions and Hypotheses

This study aims to evaluate the impact of landmark salience on drivers’ spatial cognition in autonomous driving scenarios through two simulator experiments, thereby further elucidating the mechanisms that influence spatial cognition under autonomous driving conditions. Additionally, this study will assess the impact of landmark salience on the autonomous driving takeover process. Based on previous research results (von Stülpnagel & Frankenstein, 2015), we hypothesize that salient landmarks can enhance participants’ spatial memory performance. Given that drivers under autonomous driving conditions do not need to focus intensely on driving operations (Pipkorn et al., 2022; Yang et al., 2024), they may have more cognitive resources to process road information and can pay more attention to landmarks. Therefore, we further hypothesize that salient landmarks will help expedite the takeover process, with participants completing takeover tasks in a shorter time.

The visual and structural characteristics of landmarks have been analyzed by various researchers, who concluded that both characteristics had an effect on people’s wayfinding behavior. Hence, we can assume that these characteristics are key to landmarks being used by people. Therefore, this study primarily investigates the effects of the visual and structural salience of landmarks on spatial cognition and the takeover process during autonomous driving.

### 1.4. Significance of the Study

The core question this study seeks to answer is: How can we enhance drivers’ spatial cognition and improve takeover efficiency by optimizing landmark salience design in autonomous driving scenarios? Answering this question is crucial for the safe application of autonomous driving technology and will fill gaps in existing research, as well as explore the practical application value of landmark salience in autonomous driving. Our approach integrates cognitive psychology with applied human factors design, providing novel insights that can inform both intelligent driving systems and urban road planning.

## 2. Experiment 1

The purpose of Experiment 1 was to investigate the impact of landmark salience on drivers’ spatial cognition in autonomous driving scenarios. In this study, landmark visual salience was manipulated at two levels—high and low—representing the degree to which a landmark visually stands out from its surroundings based on attributes such as color, size, contrast, and visibility. Landmark structural salience was also categorized into high and low levels, reflecting the positional significance of the landmark within the driving environment. High structural salience referred to landmarks placed at critical navigational points, such as route intersections, whereas low structural salience referred to landmarks located at location along the route.

### 2.1. Methods

#### 2.1.1. Participants

A total of 56 students from the university, aged between 18 and 26 years, participated in Experiment 1, comprising an equal number of males and females. All participants were screened using the Santa Barbara Sense of Direction (SBSOD) scale (Hegarty et al., 2002) to ensure their spatial abilities were at a moderate level, without extreme cases of either too good or too poor. In Experiment 1, we focused on spatial cognition tasks, which are traditionally considered to be influenced by an individual’s spatial abilities. By using the SBSOD scale, we were able to screen participants and select those with a balanced level of spatial ability. This screening process helped us ensure that the variability in the spatial cognition task results was due to our experimental variables (i.e., landmark visual salience and structural salience) rather than individual differences in participants’ spatial abilities. Participants were randomly assigned to one of four experimental groups and received compensation upon completion of the experiment. The Ethics Committee of the Academy of Psychology and Behavior at Tianjin Normal University approved this study. All participants signed the informed consent and voluntarily participated in the study. They were informed that they could withdraw from the study at any time without any consequences.

#### 2.1.2. Design

Experiment 1 employed a 2 (Landmark Visual salience: High, Low) × 2 (Landmark Structural salience: High, Low) between-subjects design. The dependent variable was performance on spatial cognition tasks.

#### 2.1.3. Materials

The Santa Barbara Sense of Direction (SBSOD) Scale (Hegarty et al., 2002) was used to classify participants’ spatial ability levels. This 15-item self-report scale assesses individuals’ perceived navigational and directional capabilities. Participants rate their agreement with each item on a 7-point Likert scale ranging from “strongly disagree” to “strongly agree.” Higher scores reflect better perceived sense of direction. The scale has demonstrated good psychometric properties across studies in spatial cognition.

To control the visual salience of landmarks, we conducted visual salience ratings on 45 landmark images before the formal experiment began and selected high and low visual salience landmarks from them. These 45 images included plants, sculptures, vehicles, traffic signs, urban infrastructure, buildings, and pedestrians. The color and shape proportions of the landmarks were set with reference to the actual urban road environment, and the landmarks were placed in the experimental virtual environment and captured as questionnaire rating materials.

**Figure 1:**
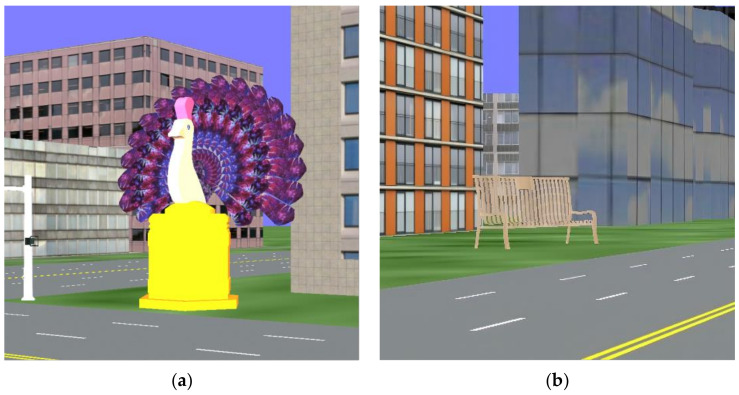
Example landmarks: (**a**) a landmark with high visual salience; (**b**) a landmark with low visual salience.

Thirty university students were recruited online to participate in the rating. Participants first watched a pre-recorded video of a real experimental scene to understand the landmarks, roads, and surrounding environment from a first-person perspective, eliminating the influence of the order of landmark appearance on the evaluation results. Participants then completed a landmark visual salience rating questionnaire; a simple definition of salience was provided before the questionnaire, guiding participants to rate the visual salience of the landmarks in the images (1 = completely not salient, 9 = completely salient). Finally, based on the average score of each image, all images were ranked by visual salience, and the top and bottom 8 landmarks were selected as high and low visual salience landmarks, respectively. A paired samples t-test was conducted to compare the perceived visual salience between high salience and low salience landmark images. The results showed a significant difference between the two image groups, *t*(7) = 63.88, *p* < 0.001, indicating that the average scores for the high salience and low salience groups were significantly different. Specifically, the high salience group had a mean score of 8.03 (*SD* = 0.25), with scores ranging from 7.70 to 8.40, while the low salience group had a mean score of 3.15 (*SD* = 0.09), with scores ranging from 3.03 to 3.30. The results indicated that participants consistently perceived substantial visual differences between the two sets of images. Figure 1 presents examples of visually salient landmarks, including one with high visual salience and another with low visual salience.

**Figure 2:**
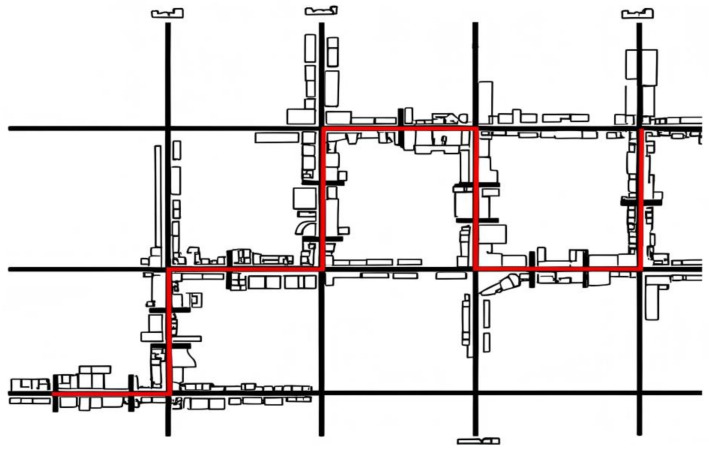
Bird’s-eye view of the road layout used in the simulation scenario. (Note. The red line represents the designated driving route).

A three-dimensional town scene containing roads and buildings was constructed using Sketchup. The town consisted of three horizontal and four vertical roads, as well as several smaller roads. All roads were bidirectional three-lane roads, 3.5 m wide, with a total route length of 8 km, including 21 intersections, seven of which required turning (see Figure 2).

**Figure 3:**
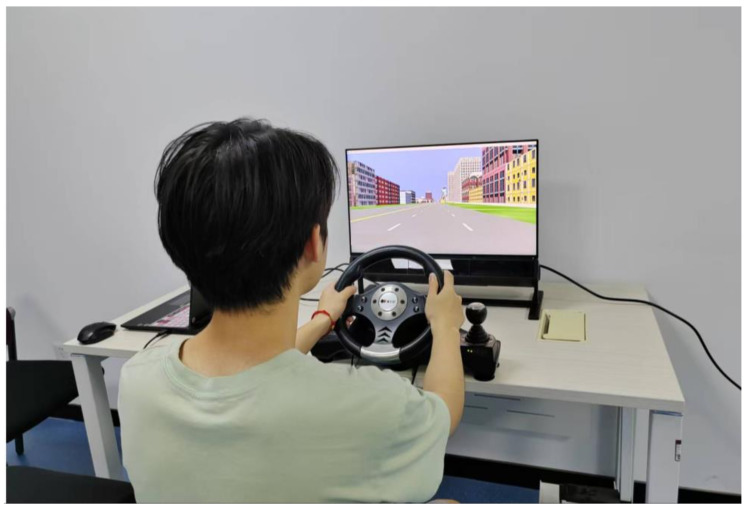
Simulation driving scenario and driving equipment used in the experiment.

The virtual environment was rendered using WorldViz Vizard software version 5.0 to achieve scene interaction. The environment was displayed on a 23-inch screen with a resolution of 1920 × 1080 pixels and a refresh rate of 75 Hz, presenting the road environment from an egocentric perspective. The vehicle had two driving modes: in manual driving mode, the driver controlled the virtual vehicle’s forward, braking, reversing, and steering activities using pedals and a steering wheel, as shown in Figure 3; in autonomous driving mode, the driver did not need to control the virtual vehicle in real-time, as it was programmed to drive autonomously. During the preparation phase, voice navigation was provided to the driver, with instructions such as “Turn right (left or straight) at the next intersection”.

**Figure 4:**
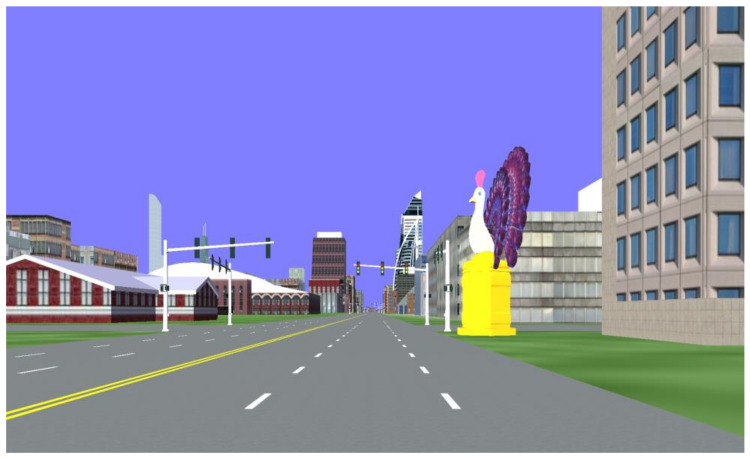
Experimental scene featuring a landmark with high visual salience located at an intersection.

All four groups of participants used the same experimental scene, with differences in the selection and placement of landmarks. Group 1 placed high-visual-salience landmarks at intersections, ensuring balance in all four directions, as shown in Figure 4. Group 2 placed low-visual-salience landmarks along the roadside, ensuring an even distribution on both sides of the road. Group 3 placed high-visual-salience landmarks along the sides of the road, ensuring an even distribution on both sides. Group 4 placed low-visual-salience landmarks at intersections, ensuring balance in all four directions.

#### 2.1.4. Procedure

The experiment took approximately 30 min. Before the experiment began, participants were required to complete a questionnaire that collected basic information, such as age, gender, and driving experience.

The experiment consisted of three stages: training, learning, and task stages, which participants completed sequentially.

In the training stage, a short simulated driving route helped participants familiarize themselves with the equipment operation. During this stage, participants needed to follow navigation prompts to manipulate the steering wheel and pedals to drive the vehicle from the starting point to the end point. They were required to follow traffic rules, not driving off the road, stopping unnecessarily, or driving in the wrong direction. Upon reaching the endpoint and seeing a sign, the stage ended. This stage was conducted only once, and the scene did not appear in subsequent tasks.

During the learning stage, participants were informed that they did not need to control the steering wheel and pedals, as the vehicle could drive autonomously; however, they were still required to keep their hands on the steering wheel and remain attentive to the driving process and the surrounding environment. This stage was conducted twice, with a one-minute interval between each.

In the task stage, participants were required to complete three spatial cognition tasks in sequence: re-cruise, scene recognition, and sequence recognition.

Re-cruise task: Participants began at the scene’s starting point and drove the vehicle to the endpoint without navigation prompts. The task scene was the same as in the learning stage. If participants deviated from the correct route during driving, the system would correct them back to the correct route after the deviation, with minimal impact on the participant’s driving. The program automatically recorded driving time and the number of incorrect intersections.

Scene recognition task: Participants were asked to determine whether the scene images displayed on the screen had appeared during the learning stage. The task included 28 images, 14 of which were scenes participants had seen in the previous learning stage (old scenes) and 14 of which were new scenes they had not encountered. Participants pressed “F” to indicate that the scene had appeared and “J” to indicate that it had not. The order of old and new scenes was balanced. The program recorded response time and feedback results for each image.

Sequence recognition task: Two scene images that had been experienced in the learning stage were simultaneously presented on the screen. Participants were asked to determine which of the two images appeared earlier, using the “F” key to indicate that the left image appeared first and the “J” key to indicate that the right image appeared first. The experiment included 30 such tasks, with half having the left image appear first and the other half having the right image appear first. The program also recorded the response time and feedback results for each task.

#### 2.1.5. Data Analysis

For the re-cruise task, given that there were two types of intersections (straight and turning), it was necessary to calculate the decision accuracy seperately for all intersections (a total of 20), turning intersections (a total of seven), and straight intersections (a total of 13) separately.

The data were analyzed using SPSS Statistics 27.0. The results of each task were analyzed separately. A 2 (Landmark Visual salience: High, Low) × 2 (Landmark Structural salience: High, Low) between-subjects ANOVA was used to evaluate the effects of visual and structural salience on the experimental outcomes. Data with values greater than or equal to ±3σ from the mean were considered outliers and excluded from the analysis. One participant exhibited a value greater than 3σ in the scene recognition task. We excluded this participant’s data from the scene recognition task. To maintain consistency across tasks, we excluded this participant’s data from all subsequent analyses. This approach ensured that all analyses were conducted on a consistent participant set. As a result, the final sample included valid data from 55 participants.

### 2.2. Results

**Figure 5:**
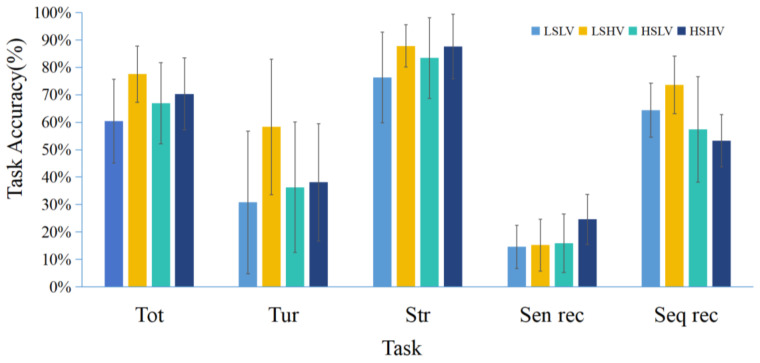
Task accuracy across different experimental conditions for each task type. (Note. LS = low structural, HS = high structural, LV = low visual, HV = high visual. LSLV refers to landmarks located along the route with low visual salience; LSHV refers to landmarks located along the route with high visual salience; HSLV refers to landmarks located at intersections with low visual salience; HSHV refers to landmarks located at intersections with high visual salience. Tot refers to the decision accuracy for all intersections; Tur refers to the decision accuracy for turning intersections; Str refers to the decision accuracy for straight intersections; Sen rec refers to the scene recognition accuracy; Seq rec refers to the sequence recognition accuracy.

Figure 5 illustrates the accuracy rates of different groups across various tasks. The experimental results will be described in detail subsequently.

#### 2.2.1. Re-Cruise Task

The results indicate a main effect of visual salience on accuracy at all intersections (*F*(1,51) = 7.458, *p* = 0.009, *η^2^* = 0.128). Landmarks with high visual salience (*M* = 0.732, *SD* = 0.124) lead to higher accuracy compared to those with low visual salience (*M* = 0.638, *SD* = 0.154) at all intersection decisions. The main effect of structural salience (*F*(1,51) = 0.009, *p* = 0.924) and the interaction effect (*F*(1,51) = 3.365, *p* = 0.072) were not significant. For accuracy at turning intersections, there was a main effect of visual salience (*F*(1,51) = 4.607, *p* = 0.037, *η*^2^ = 0.083). Landmarks with high visual salience (*M* = 0.462, *SD* = 0.245) led to higher accuracy compared to those with low visual salience (*M* = 0.343, SD = 0.247). The main effect of structural salience (*F*(1,51) = 1.524, *p* = 0.223) and the interaction effect (*F*(1,51) = 3.481, *p* = 0.068) were not significant. For accuracy at straight intersections, there was a main effect of visual salience (*F*(1,51) = 5.188, *p* = 0.027, *η*^2^ = 0.092). Landmarks with high visual salience (*M* = 0.877, *SD* = 0.102) led to higher accuracy compared to those with low visual salience (*M* = 0.797, *SD* = 0.160) in intersection decisions. The main effect of structural salience (*F*(1,51) = 1.114, *p* = 0.296) and the interaction effect (*F*(1,51) = 1.243, *p* = 0.270) were not significant.

#### 2.2.2. Scene Recognition Task

The results show a significant main effect of structural salience (*F*(1,51) = 4.432, *p* = 0.040, *η*^2^ = 0.080), with landmarks at decision points (*M* = 0.210, *SD* = 0.106) leading to higher accuracy compared to those located at non-decision points (*M* = 0.149, *SD* = 0.085) in scene recognition. The main effect of visual salience (*F*(1,51) = 3.280, *p* = 0.076) and the interaction effect (*F*(1,51) = 2.490, *p* = 0.121) were not significant. Although landmarks with high visual salience (*M* = 0.208, *SD* = 0.103) showed higher accuracy than those with low visual salience (*M* = 0.153, *SD* = 0.093) in the scene recognition task, the difference was not statistically significant.

#### 2.2.3. Sequence Recognition Task

The results indicate a significant main effect of structural salience, *F*(1,51) = 14.351, *p* < 0.001, *η*^2^ = 0.220. Landmarks at non-decision points (*M* = 0.686, *SD* = 0.112) led to higher accuracy compared to those located at decision points (*M* = 0.551, *SD* = 0.143) in sequence recognition. The main effect of visual salience was not significant, *F*(1,51) = 0.713, *p* = 0.402. Although landmarks with high visual salience (*M* = 0.614, *SD* = 0.141) showed higher accuracy than those with low visual salience (*M* = 0.604, *SD* = 0.155) in the sequence recognition task, the difference was not statistically significant. The interaction effect (*F*(1,51) = 4.079, *p* = 0.049), *η*^2^ = 0.074 was significant, simple effects analysis revealed that under high visual salience landmark conditions, landmarks at non-decision points (*M* = 0.736, *SD* = 0.105) yielded higher accuracy than landmarks at decision points (*M* = 0.533, *SD* = 0.096) in the sequence recognition task (*F*(1,51) = 18.164, *p* < 0.001, *η*^2^ = 0.263).

### 2.3. Discussion

Experiment 1 explored the impact of landmark visual salience and structural salience on drivers’ spatial cognition in autonomous driving scenarios through three task types. The results indicated that these two types of landmark salience had different effects across various task types.

In the re-cruise task, visual salience had a significant main effect on decision accuracy at all intersections, turning intersections, and straight intersections. Specifically, landmarks with high visual salience significantly improved participants’ accuracy in intersection decisions, consistent with the findings of Denis et al. (2014), who discovered that rich visual landmarks aid route memory. This result suggested that visually prominent landmarks provide clearer navigation cues in complex intersection environments. However, structural salience and its interaction with visual salience did not show significant effects in any task type, indicating that structural salience may play a limited role in the re-cruise task.

In the scene recognition task, structural salience showed a significant main effect, indicating that landmarks at decision points significantly improved participants’ scene recognition accuracy, consistent with research on pedestrian navigation (Bruns & Chamberlain, 2019). Given that drivers in autonomous scenarios do not need to focus on driving operations, they have more attentional resources with which to process environmental information, making landmarks at decision points more likely to be noticed and remembered. However, visual salience and the interaction effect did not reach significance, suggesting that structural salience played a more critical role in the scene recognition task, while the importance of visual salience was relatively lower. The reason for this effect might be that highly salient landmarks catch attention from the other scenes right before and after this scene. Participants learned a list of scenes. A salient landmark leads to better recognition of this landmark, but the recognition rate of the scenes just before and after this salient landmark decreases. This phenomenon is consistent with findings from attentional capture research (Theeuwes, 1994; Itti & Koch, 2000), where highly salient stimuli tend to draw attention away from surrounding elements, a process sometimes referred to as peripheral suppression.

In the sequence recognition task, structural salience also showed a significant main effect; surprisingly, landmarks at non-decision points significantly improved sequence recognition accuracy. This result further supported the importance of structural salience in cognitive memory tasks and emphasized that landmarks at non-decision points are also crucial for navigation.

Overall, Experiment 1 found that landmark visual salience played a significant role in practical navigation tasks (such as the re-cruise task), while structural salience played a more important role in cognitive memory tasks (such as scene recognition and sequence recognition tasks). This suggests that landmark visual salience and structural salience play different roles in different types of cognitive tasks. Nevertheless, the results showed that salient landmarks in the environment helped improve drivers’ spatial cognition performance in autonomous driving scenarios. In Experiment 2, we aimed to further explore the impact of landmark salience on drivers’ takeover performance.

## 3. Experiment 2

The purpose of Experiment 2 was to investigate the impact of landmark salience on drivers’ takeover performance in autonomous driving scenarios. As in Experiment 1, landmark visual salience was manipulated at two levels: high and low. Similarly, landmark structural salience was also categorized as high or low.

### 3.1. Methods

#### 3.1.1. Participants

A total of 56 students from the university, aged between 18 and 26 years, participated in Experiment 1, comprising an equal number of males and females. All participants were screened using the Santa Barbara Sense of Direction (SBSOD) scale (Hegarty et al., 2002) to ensure their spatial abilities were at a moderate level, without extreme cases of either too good or too poor. Consistent with Experiment 1, we applied the same participant screening procedure to Experiment 2 to ensure that any variation in takeover performance was due to our independent variables (i.e., landmark visual salience and structural salience) rather than individual differences in spatial ability. Participants were randomly assigned to one of four experimental groups, with the grouping identical to that in Experiment 1. The Ethics Committee of the Academy of Psychology and Behavior at Tianjin Normal University approved this study. All participants signed the informed consent and voluntarily participated in the study. Participants received some compensation after the experiment.

#### 3.1.2. Design

Experiment 2 employed a 2 (Landmark Visual Salience: High, Low) × 2 (Landmark Structural Salience: High, Low) between-subjects design. The dependent variable was takeover time.

#### 3.1.3. Materials

The materials and experimental conditions were consistent with those in Experiment 1. The experimental scenario was the same as in Experiment 1, but a takeover obstacle was placed on the road where each landmark appeared. There were four types of takeover obstacles: pedestrians, traffic accidents, traffic barriers, and road blur. Pedestrians walked upright across the road, obstructing the vehicle’s forward direction. Traffic accidents involved two cars rear-ending each other. Traffic barriers were common yellow circular triangle pillars arranged horizontally to block the vehicle’s path. Road blur was achieved by eliminating the road markings, requiring participants to judge the road’s forward direction themselves. The takeover tasks were located slightly to the left of the road’s center, providing participants with ample space to complete the takeover. A voice prompt, “Please take over the vehicle,” was continuously played during the takeover process. The procedure recorded the time between the participant’s start and end takeover button presses to calculate the takeover completion time.

#### 3.1.4. Procedure

Based on the preparation phase of Experiment 1, Experiment 2 added a takeover scenario. Before the preparation phase began, participants were thoroughly instructed on the takeover process to ensure they could complete the takeover smoothly after the preparation phase.

In the formal experiment, participants initiated the autonomous driving mode by pressing a button while keeping their hands on the steering wheel. During the automated drive, a takeover obstacle appeared on the road, accompanied by a voice prompt instructing, “Please take over the vehicle.” The takeover signal had a lead time of approximately 7 s. Upon hearing the prompt, participants began the takeover operation by pressing a designated button and pressing it again upon completing the maneuver. After the takeover was completed, the system played a second prompt, “Entering autonomous mode,” indicating the resumption of automated control. Participants were required to keep their hands on the steering wheel throughout, although no manual adjustments to steering or speed were necessary during automated driving. A total of eight takeover events occurred during the driving session, each triggered at the location of a landmark positioned at an intersection. The timestamps of each button press were recorded to measure takeover duration.

#### 3.1.5. Data Analysis

The time interval between the two button presses was used to measure the takeover time. Each participant needed to complete eight takeovers. The mean duration of these eight takeovers was the participant’s takeover duration indicator.

The data were analyzed using SPSS Statistics 27.0. A 2 (Landmark Visual salience: High, Low) × 2 (Landmark Structural salience: High, Low) between-subjects ANOVA was used to evaluate the effects of visual and structural salience on takeover time. Data from three participants who did not complete the entire experiment were excluded from the analysis. We were ultimately able to collect complete data from 53 participants.

### 3.2. Results

**Figure 6:**
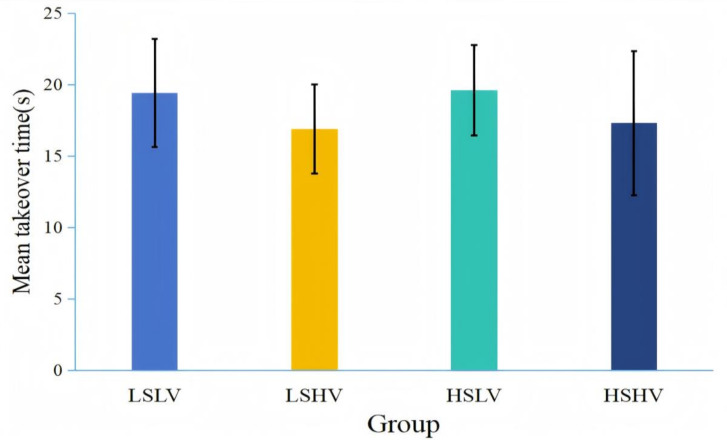
Takeover time across different experimental conditions. (Note. LS = low structural salience; HS = high structural salience; LV = low visual salience; HV = high visual salience. LSLV refers to landmarks located along the route with low visual salience; LSHV refers to landmarks located along the route with high visual salience; HSLV refers to landmarks located at intersections with low visual salience; HSHV refers to landmarks located at intersections with high visual salience).

Figure 6 illustrates the takeover time of different groups. The results indicated a significant main effect of visual salience (*F*(1,49) = 5.072, *p =* 0.029, *η*^2^ = 0.094), with landmarks of high visual salience (*M* = 17.098, *SD* = 4.150) resulting in shorter takeover time than the landmarks of low visual salience (*M* = 19.504, *SD* = 3.462). The main effect of structural salience (*F*(1,49) = 0.083, *p* = 0.775) and the interaction effect (*F*(1,49) = 0.010, *p* = 0.920) were not significant.

### 3.3. Discussion

Experiment 2 demonstrated that visually salient landmarks had a significant influence on takeover time. Specifically, landmarks with high visual salience significantly shortened takeover time, supporting our hypothesis.

Previous research has shown that using partially automated vehicles on highways could easily lead drivers to become bored and less vigilant (McWilliams & Ward, 2021). During autonomous driving, drivers tended to reduce their attention to the road ahead and instead focus on NDRT or their surroundings (Pipkorn et al., 2022; Yang et al., 2024), thereby weakening their situational awareness (Xie et al., 2022). In this context, introducing landmarks with high visual salience could effectively enhance individuals’ perception of environmental changes, enabling them to react more quickly. However, the impact of structural salience on takeover duration was not significant, suggesting that in such complex situations, participants may have found it difficult to distinguish between decision-point landmarks and non-decision-point landmarks. An explanation for this might be that takeover scenarios are not related to decision points, and therefore, the structural salient landmarks had no meaning.

## 4. General Discussion

This study explored the impact of landmark salience on drivers’ spatial cognition and takeover performance in autonomous driving scenarios through two experiments. Experiment 1 assessed the influence of landmark salience on drivers’ spatial cognition, while Experiment 2 further examined the impact of landmark salience on drivers’ takeover time.

The results of Experiment 1 indicated that visually salient landmarks significantly influenced drivers’ decision accuracy at intersections during the re-cruise task. Landmarks with high visual salience significantly improved decision accuracy at all types of intersections. The re-cruise task was considered a typical egocentric navigation task, where spatial information was encoded from the navigator’s perspective (Wolbers & Wiener, 2014). These results suggested that visually attention-grabbing landmarks could more effectively help drivers encode spatial information and construct route knowledge, guiding them to make correct intersection choices in autonomous driving scenarios. In contrast, structural salience had no significant impact on decision accuracy, showing neither an independent main effect nor an interaction with visual salience. This indicated that while the structural salience of landmarks might influence drivers’ spatial cognition in certain other contexts, such as memory tasks, visual salience appeared to play a more critical role in the re-cruise task. The cognitive load theory posits that human cognitive resources are limited (Sweller et al., 2019). In complex driving environments, drivers must process a large amount of visual and spatial information to make accurate navigation decisions. At this point, drivers allocated their limited cognitive resources to the most important environmental cues, such as landmarks, to effectively construct spatial memory and make correct route choices. Previous research had shown that visually salient landmarks were more likely to attract individuals’ attention (Wenczel et al., 2017) and enhance route memory (Denis et al., 2014). High-visual-salience landmarks could quickly capture drivers’ attention, reducing cognitive load on other less important information and enabling the more efficient allocation of cognitive resources to these key landmarks. Therefore, in the re-cruise task, high-visual-salience landmarks served as significant navigation cues, helping drivers encode and remember routes more quickly, thereby improving task accuracy. Additionally, since the re-cruise task primarily required drivers to form an overall memory of routes and landmarks rather than precisely identify individual decision points, structural salience (typically manifested in landmarks located at critical decision points or turns) had a smaller direct impact on this task.

In the scene recognition and sequence recognition cognitive memory tasks, structural salience showed a significant main effect, indicating that landmarks at decision points could improve drivers’ scene recognition accuracy, while landmarks at non-decision points significantly enhanced sequence recognition accuracy. However, visual salience did not show a significant impact. It is worth noting that although the main effect of visual salience was not significant, descriptive statistics (group means and standard deviations) revealed a trend of higher accuracy with high-visual-salience landmarks, indicating that visually salient landmarks also played a certain role but were not critical. They were more suitable for helping with timely attention allocation and perception tasks, such as the re-cruise task. In the cognitive memory tasks, individuals were required to recall specific scenes or sequences of events after a specified period. These tasks focused on how individuals extracted and stored meaningful spatial information from their environment, which was often closely related to decision points. Consistent with previous emphasis on the importance of landmarks at turning intersections (Janzen, 2006; Janzen & van Turennout, 2004), structurally salient landmarks, located at critical decision points (such as turns or route bifurcations), served as important reference points in spatial memory, aiding in the identification of paths and critical decisions. As previously emphasized, landmarks at non-decision points were also important (Lovelace et al., 1999; Tlauka & Wilson, 1994). Even if they were not located at decision points, they still helped individuals remember paths or scenes because they provided visual reference points, assisting individuals in spatial orientation.

Building on Experiment 1, Experiment 2 further explored the impact of landmark salience on drivers’ takeover performance. The results of Experiment 2 indicated that visual salience had a significant influence on takeover reaction time. Specifically, landmarks with high visual salience significantly shortened drivers’ takeover duration. This result further emphasized the importance of enhancing the visual salience of landmarks to improve drivers’ takeover efficiency in autonomous driving scenarios. Similar to experiment 1, the impact of landmark structural salience on takeover reaction time was not significant, and there was no significant interaction with visual salience. This result indicated that, in the decision task of takeover, visual salience remained the most critical factor. Given the general characteristics of reduced attention and vigilance among drivers in autonomous driving scenarios (Greenlee et al., 2018; Pipkorn et al., 2022; Tivesten et al., 2019; Victor et al., 2005). For traditional driving, roadside advertisements could easily attract drivers’ attention, causing distraction and adversely affecting their driving performance (Megías et al., 2011; Walker & Trick, 2019). However, for drivers in autonomous vehicles, this distraction might be beneficial to some extent, as it could help them overcome boredom and speed up the recovery of situational awareness, increasing the likelihood of detecting dangerous situations.

In summary, both experiments consistently demonstrated that the visual salience of landmarks played an important role in drivers’ spatial cognition and takeover behavior. In autonomous driving scenarios, landmarks with high visual salience not only improved the accuracy of intersection decisions but also shortened drivers’ takeover reaction time. Structural salience of landmarks, on the other hand, had a greater impact on scene recognition and sequence recognition tasks. However, its influence on dynamic decision tasks (such as takeover reaction time) was weaker. These findings have significant implications for future urban road planning. In road design, visually salient landmarks should be emphasized, especially at complex intersections and critical decision points, where high-visual-salience landmarks could help drivers more accurately identify intersections and react more quickly and effectively when manual takeover is needed.

While the present study highlights the positive effects of salient landmarks on spatial cognition and takeover performance, it is important to recognize that salience is inherently a relative concept. However, if the number of salient landmarks increases, the salience of individual landmarks decreases because there are many other salient landmarks. Therefore, the goal should not be to simply increase the number of salient landmarks to enhance drivers’ spatial cognition and takeover performance, but rather to determine the optimal number and distribution that maximizes their effectiveness. Therefore, the goal cannot be to simply increase the number of salient landmarks but to find the optimal amount. There may be a trade-off between the number of salient landmarks and their individual effectiveness. This will be particularly important for urban planning and the design of autonomous driving interfaces.

Our study had some limitations. First, the selection of landmarks was based on real environments, and the assessment of visual salience was subjective, making this evaluation method relatively crude. Future research could consider using eye-tracking data to construct landmark salience models (Wang et al., 2020). Second, eye-tracking technology has been widely used in the field of autonomous driving, enabling more accurate measurement of drivers’ attention levels and cognitive load (Broadbent et al., 2023; Liang et al., 2021). In further research, relevant technologies could be considered. Finally, due to the complexity of real driving conditions, accurately reproducing real-world driving experiences in virtual environments was very challenging, so caution was needed when generalizing the results of virtual environment studies to the real world.

## 5. Conclusions

The present study demonstrates the influence of landmark salience on drivers’ spatial cognition and takeover performance in autonomous driving scenarios. While salient landmarks generally enhance spatial cognition, the effects of visual and structural salience differ in scope and function in autonomous driving scenarios. Landmarks with high visual salience not only improved drivers’ accuracy in making intersection decisions but also significantly reduced takeover reaction time. In contrast, structurally salient landmarks had a more pronounced effect on memory-based tasks, such as scene and sequence recognition, but showed limited influence on dynamic decision-making tasks like takeover response. These findings underscore the differentiated roles of visual and structural landmark features and highlight the critical importance of visually salient cues in supporting both navigation and timely takeover during autonomous driving.

## Data Availability

The datasets generated during and/or analyzed during the current study are available from the first author upon reasonable request.

## Abbreviations

The following abbreviations are used in this manuscript:

AVs	Autonomous vehicles
NDRTJ	Non-driving-related tasks
(TOR)	Take-Over request
GPS	Global Positioning System
LSLV	Low structural, Low visual
LSHV	Low structural, High visual
HSLV	High structural, Low visual
HSHV	High structural, High visual
